# Complicated sinusitis with sphenopalatine artery thrombosis in a COVID-19 patient: a case report

**DOI:** 10.1093/jscr/rjab010

**Published:** 2021-03-08

**Authors:** Omar Ahmed, Youssef Aladham, Sara Mahmood, Moustafa Mohamed Abdelnaby

**Affiliations:** Department of Otolaryngology, East Kent Hospitals University NHS Foundation Trust, Kent, UK; Department of Otolaryngology and Head and Neck Surgery, University Hospitals of Derby and Burton NHS Foundation Trust, Derby, UK; Department of Otolaryngology, University Hospitals of Leicester NHS Trust, Leicester, UK; Department of Otolaryngology, Alexandria University, Midan al Khartoum, Alexandria, Egypt

**Keywords:** COVID-19, complicated sinusitis, palatal necrosis

## Abstract

COVID-19 has been a mystery against healthcare professionals. We herein report a rare presentation of complicated sinusitis with pre-septal cellulitis and hard palatal necrosis in a COVID-19 patient. A 52-year-old male was admitted to the hospital with typical COVID manifestations where he had two successive COVID-19 positive swabs. During his admission, he developed symptoms of right orbital complications of sinusitis along with both clinical and radiological evidence of ipsilateral hard palatal necrosis. Imaging confirmed a diagnosis of right pan-sinusitis complicated with right pre-septal infection and hard palatal bony defect on the same side. Our case focuses on the possible association between these manifestations and the known thromboembolic complications of COVID-19. Ongoing management of such complicated rare cases should be through multidisciplinary team.

## INTRODUCTION

The World Health Organization recognized the novel corona virus (SARS-CoV-2) as a cause of respiratory infectious pandemic in early 2020 [1]. Comprehensive understanding of its full scale would enable us to mitigate its spread and manage its complications. There have been a few reports in literature where complicated sinusitis has occurred in association with COVID-19 infections [2]. Complicated sinusitis is generally encountered more commonly among immunocompromised patients. We present a case report which highlights the possible correlation between the hypercoagulable state of COVID-19 and the occurrence of orbital complications and palatal necrosis in patients with complicated COVID-19-induced sinusitis.

## CASE REPORT

A 52-year-old male presented to the Accident and Emergency Department with a 5-day history of high-grade fever, rhinorrhea, dry cough which is worse at night and an altered sense of smell. His symptoms progressed over the following 48 hours to include dyspnea and generalized myalgia. He is a well-controlled type 2 diabetic patient and is otherwise fit and well.

Initial assessment showed a heart rate of 95 beats per minute, respiratory rate of 28/minute, a temperature of 38.6°C and a blood pressure of 130/85 mmHg. His oxygen saturation was 85% on room air, for which high-flow oxygen was provided. Bilateral basal crepitations were heard on chest auscultation. A diagnosis of COVID-19 was suspected; hence, nasopharyngeal and oropharyngeal swabs were sent followed by a prompt transfer to an isolation room.

His laboratory investigations showed a normal full blood count. Blood D-dimer level was 7.15 mg/l (reference range = 0.15–0.45) and fibrinogen was 5.68 g/l (reference range = 1.50–4.50). Plasma procalcitonin was normal, ruling out possible bacterial aetiology. A computed tomography (CT) of the chest revealed bilateral patchy opacifications in both upper and lower zones of the lungs. Prophylactic dose of enoxaparin was commenced. On the following day, his COVID-19 swab turned out to be positive.

**Figure 1:**
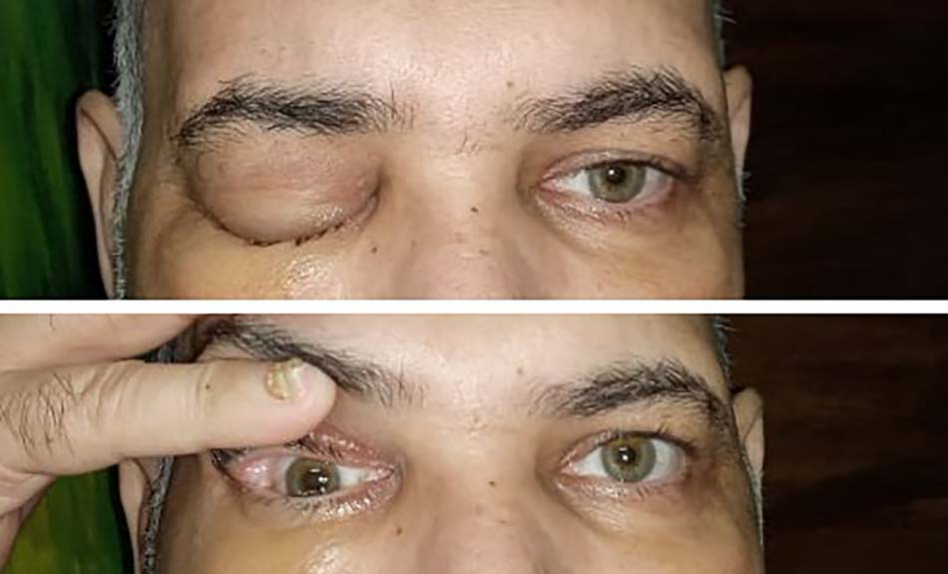
Periorbital oedema of the right eyelids; note the absence of chemosis and proptosis.

**Figure 2:**
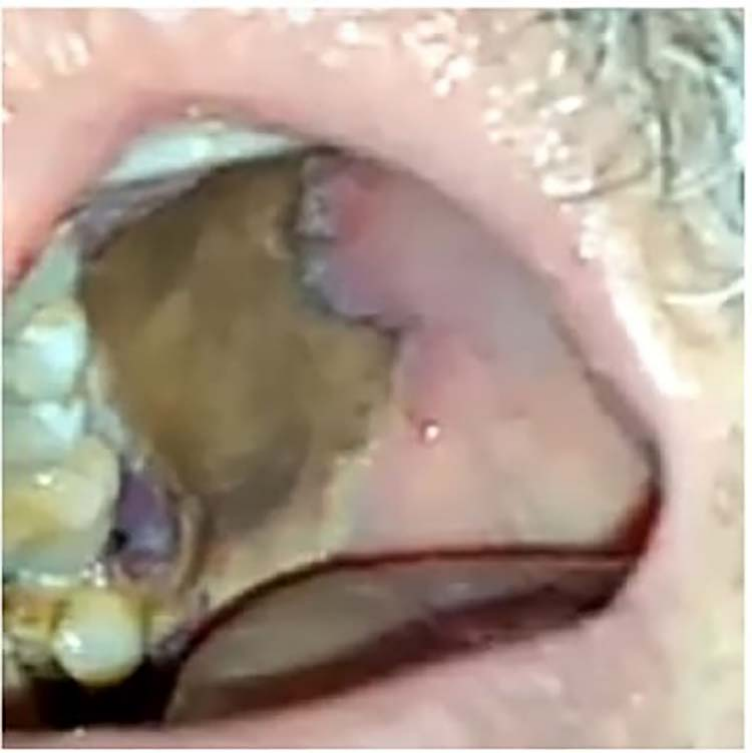
Oral cavity view showing extensive necrosis of the right palate.

The patient remained stable and was maintained on high-flow oxygen. A second COVID-19 swab after 72 hours remained positive. Two days later, he developed a new onset of paresthesia and swelling of the right eyelids and cheek, worsening headache and persistent right nasal blockage. Nasendoscopy revealed brown discharge in the right nasal cavity while the left side was unremarkable. Ophthalmological examination revealed features of preseptal cellulitis with periorbital oedema involving both upper and lower eyelids (Fig. 1). The patient had normal visual acuity bilaterally with no ophthalmoplegia, chemosis or proptosis. Oropharyngeal examination showed evidence of necrosis of the right half of the hard palate (Fig. 2).

**Figure 3:**
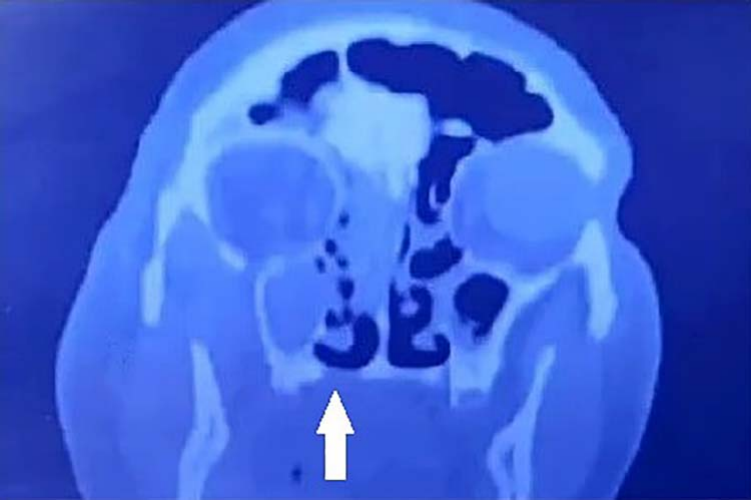
Coronal CT showing bony erosion of the right side of the hard palate (white arrow) with ipsilateral maxillary and ethmoid opacification.

Contrast-enhanced CT of the sinuses was requested on an urgent basis which showed a picture of right-sided pansinusitis with only mucosal thickening of the left maxillary sinus. In addition, there was radiological bony erosion of the floor of nose on the right side (Fig. 3). No orbital involvement was noted.

**Figure 4:**
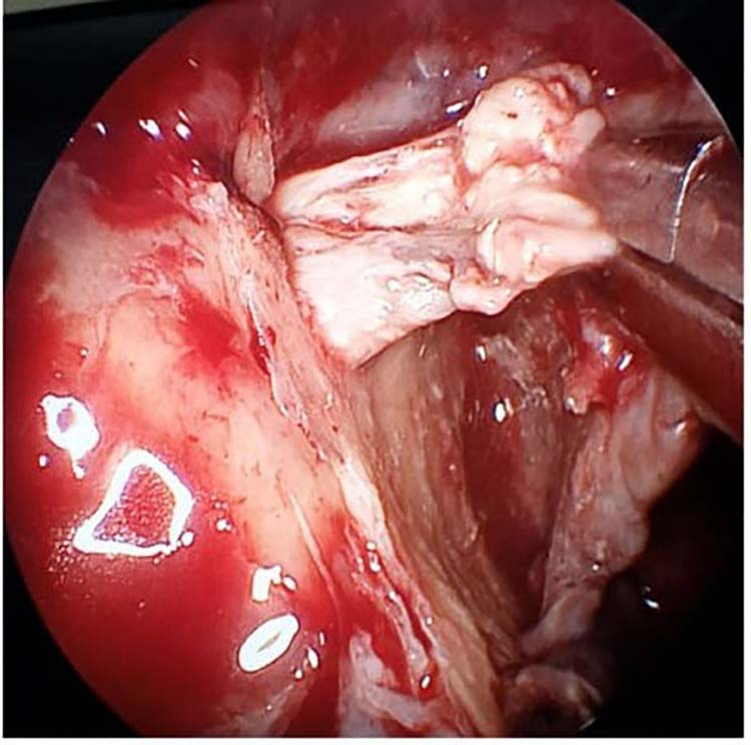
Intraoperative endoscopic image of the thrombosed right sphenopalatine artery.

After multidisciplinary discussion, he was taken to the theatre the following day where he underwent right ethmoidectomy, uncinectomy and wide middle meatal antrostomy. The right sphenopalatine artery was found thrombosed (Fig. 4). This was combined with transoral resection of the right palatine process of maxilla. A sample of the discharge was sent for bacteriologic and fungal smear and culture. Immediate palatal reconstruction was performed using a temporary obturator which was replaced 2 weeks later by a permanent one. The smear was negative for both bacterial and fungal elements, and no growth was reported in the culture. The patient’s general condition improved and was discharged 4 days later.

## DISCUSSION

COVID-19 has been shown to be a multi-systemic disease rather than just a respiratory system infection. One of its main pathophysiological mechanisms is the occurrence of a generalized pro-thrombotic state with resultant microvascular and macrovascular thromboembolism. Han *et al.* noted that coagulation parameters such as raised D-dimers, fibrin degradation products and fibrinogen correlate with the severity of COVID-19 infection. Also, these parameters were noted to be elevated in milder forms of the disease compared with healthy controls [3]. The mechanism of thrombophilia in SARS-CoV-2 infection is not fully elucidated; however, some putative mechanisms were proposed, including a pro-coagulable cytokine storm, viral tropism for angiotensin converting enzyme 2 (ACE2) receptors in vascular endothelium and raised antiphospholipid antibodies. Reports of pulmonary embolisms, deep vein thrombosis, cerebral infarction and cerebral venous sinus thrombosis associating COVID-19 infection are increasing in recent literature [4]. In our patient, we have found the right sphenopalatine artery completely thrombosed intraoperatively, and we presume that the right internal maxillary artery, its descending palatine branch or both have also been thrombosed explaining the patient’s extensive hard palate necrosis.

Despite the tropism of COVID-19 to both upper and lower respiratory mucosa, a very limited number of reports have studied its association with the development of complicated sinusitis. Turbin *et al.* reported two cases of COVID-19-associated complicated sinusitis with orbital cellulitis. One case developed along a sub-periosteal collection, while the other demonstrated superior ophthalmic vein thrombophlebitis. In both patients, bacterial and fungal smears and cultures remained repeatedly negative [5]. Similarly, our reported patient showed negative bacterial and fungal culture and smear. Albeit, it is uncertain whether SARS-CoV-2 was the sole cause of sinusitis or a contributing factor, the temporal association, normal procalcitonin level and negative bacteriologic and fungal cultures suggest a major role of the virus in its development.

Oral manifestations of COVID-19 have also sporadically reported. Since SARS-CoV-2 can infect and replicate in oral keratinocytes and fibroblasts, oral ulcerations and superficial necrosis have been described in the tongue, lips, palate and oropharynx [6]. Palatal petechiae and erosions were also described [7]. To our knowledge, this is the first case of palatal necrosis and perforation associating COVID-19 infection. We believe that a hypercoagulable state together with active sinusitis have both contributed to its development.

We present this case hoping that it may add to the evolving understanding of the spectrum of clinical manifestations and complications of the novel pandemic.

## CONFLICT OF INTEREST STATEMENT

The authors state no conflict of interest with regard to the publication of this paper.

## FUNDING

The authors state no funding source for this work.
